# Epidemiology of burns and scalds in children presenting to the emergency department of a regional burns unit: a 7-year retrospective study

**DOI:** 10.1186/s41038-016-0047-7

**Published:** 2016-06-21

**Authors:** Ceri Elisabeth Battle, Vanessa Evans, Karen James, Katherine Guy, Janet Whitley, Phillip Adrian Evans

**Affiliations:** 1 Department of Emergency Medicine, NISCHR Haemostasis Biomedical Research Unit, Morriston Hospital, Swansea, UK; 2 Department of Physiotherapy, Morriston Hospital, Swansea, UK; 3 Department of Emergency Medicine, Morriston Hospital, Swansea, Wales SA6 6NL UK

**Keywords:** Burns epidemiology, Paediatrics, Emergency department

## Abstract

**Background:**

Variation in the incidence and mechanism of thermal injury has been reported in different age groups in children. The aim of this study was to report the incidence, mechanisms, and environmental factors of all burns presentations to the emergency department (ED) of a regional burns centre over a 7-year period.

**Methods:**

A retrospective, chart review study of all burns presentations to the ED of a regional burns centre in South Wales was conducted. All children recorded as having sustained a burn or scald, aged less than 16 years were included in the analysis. Subjects’ demographics were analysed using descriptive statistics, and comparisons were made between patients aged less than 5 and patients aged 5–16 using chi-square test and Mann–Whitney *U* test.

**Results:**

A total of 1387 cases were included in the final analysis. Scalds were the most common thermal injury with 569 (41.0 %) reported, followed by contact burns in 563 (40.6 %) patients. The patients requiring hospitalisation were significantly younger (2 vs 3 years; *p* < 0.001) and had a higher rate of non-accidental injury (10 vs 4; *p* < 0.001). The most commonly injured site in both age groups was a hand or digit.

**Conclusions:**

Scalds and contact burns were the most commonly reported thermal injury in children aged less than 16. Common mechanisms were hot beverages, hobs and hair straighteners, highlighting further burn prevention strategies are needed and good-quality prospective studies that investigate the effectiveness of such strategies.

## Background

It is estimated that 25,000 children attend emergency departments (ED) in England and Wales per year with a burn or scald, with 3800 of these needing admission for hospital care [1]. In European hospitals, children are reported to account for almost half of all burns and scalds [2]. Burn injury can lead to significant morbidity and mortality, including both physical and psychological sequelae, with a considerable associated health-economic impact [1–3].

There are a number of factors that have been demonstrated to directly influence the extent and pattern of burn injury sustained by the child, including the characteristics of the child (such as age and ethnicity), the heat source (iron, chemical agent or hot beverage), the injury mechanism (pull down or spill) and the environment in which the injury occurs (home or school, time of day the injury occurred and levels of social deprivation) [4, 5].

A recent study reported that the mechanisms, agents and patterns of burns are significantly different between children aged 5 or older and those aged less than 5 [1]. The rate of burns to the chest and face was significantly higher in the children aged less than 5 years, with older children sustaining a higher rate of outdoor injuries [1]. The most common age for a child to suffer a burn injury is 1 year, with ten times as many burns and scalds as any school year age group. The most common causes of contact burns were reported to be irons, oven hobs and hair straighteners [1].

Severity, pattern and distribution of childhood burns are influenced by the domestic environment. Contact burns from hobs, oven doors, irons and hair straighteners are increasingly reported in the paediatric population [1, 6–9]. Social deprivation has been demonstrated to be a risk factor for childhood accidents and deaths [10–12]. The aim of this study was to report the incidence, mechanisms and environmental factors of all burns presentations to the ED in a regional burns centre over a 7-year period.

## Methods

### Setting

A retrospective, single-centre study of all burns presentations to the ED of a regional burns centre in South Wales was conducted. Morriston Hospital in Swansea has over 90,000 presentations to the ED per year and serves a population of approximately 450,000. The Welsh Centre for Burns and Plastic Surgery is the regional adult burn centre for Wales and the South West of England, which serves a population of approximately ten million. Those patients coded as ‘thermal injury’ were identified using the hospital database. This study only included patients presenting directly to the ED and not those transferred directly to the burns unit. All children recorded as having sustained a burn or scald, aged less than 16 years, with both accidental and non-accidental injuries were included in the analysis. Children were divided into school (5–16 years) and non-school age (<5 years) for the analysis. Children suffering smoke inhalation only were excluded.

It was confirmed that ethical approval was not required for this study (Wales REC 6).

### Data collection

The medical notes were reviewed following guidelines suggested in a study by Gilbert et al. [13]. These guidelines included the use of standardised data extraction forms, uniform handling of ambiguous data and regular meetings between the research team to ensure standardisation of data extraction. All patients aged 16 years and under presenting to the ED of Morriston Hospital in 2008 to 2014 were examined and data recorded on a pre-designed database. A validation check was completed in which an additional researcher checked the accuracy of data input for 10 % of all patients, in order to reduce information bias. If a patient’s notes had missing or incomplete data for the variables under investigation, they were still included in the database.

The dataset included demographic variables such as age, sex, type of injury, percentage total burn surface area (TBSA) estimation, injury mechanism, injury agent, whether the injury was recorded as non-accidental, where the injury occurred (home or other) and primary anatomical area injured (according to the medical records). Outcomes recorded included mortality, hospital admission and hospital length of stay. Mechanism of injury for scald was further categorised into hot beverages (all hot drinks), domestic water (kettle, saucepan, bath, shower, tap, hot water bottle, flask, facial steamer and bowl of water) and food item (soup, cooking oil, pot noodle, gravy, curry and sauce) [1]. Contact burn mechanisms were further categorised as portable household agents (iron, hair straighteners/tongs, light bulb, saucepan, oven tray, cigarette), fixed household items (oven, hob, grill, radiator, heated pipe, fireplace/wood burner and steam press), outdoor items (exhausts, firework, BBQ, bonfire, sand) and miscellaneous (not fitting other categories) [1]. Chemical burns were further categorised as spills, ingestion, and eye splash. Radiation burns refer to thermal injury caused directly by the sun or sunbeds.

To ensure confidentiality, patients’ names were not recorded during the data collection period. The dataset was also stored on a hospital encrypted computer to ensure data security (safe-end protector encryption).

### Data analysis

Statistical analyses were performed using SPSS Version 21 (Chicago). Subjects’ demographics were analysed using descriptive statistics including numbers and percentages for categorical data and medians and interquartile ranges for continuous data (due to non-normal distribution). Comparisons were made between patients aged less than 5 and patients aged 5–16 using chi-square test (categorical variables) and Mann-Whitney *U* test (continuous variables). A two-sided probability value less than 0.05 was considered significant.

## Results

Between 2008 and 2014, a total of 1459 children were identified on the hospital database. A total of 23 (1.6 %) patients were excluded as they were not with a burn or scald, the medical records of 23 (1.6 %) patients could not be located and 26 (1.8 %) were excluded as they had sustained a smoke inhalation only. A total of 1387 cases were included in the final analysis. The median age of the included patients was 2 (IQR, 1–8), with 802 (57.8 %) patients recorded as male. A total of 1192 (85.9 %) of all burn injuries occurred in the patient’s own home. Non-accidental injury was suspected in a total of 14 (1.0 %) of all included patients and deliberately self-inflicted burns in a total of 6 (0.4 %) patients. The estimated TBSA was only documented in 876 (63.2 %) of patients, with a median of 1 % (IQR, 1–2).

Scalds were the most common thermal injury with 569 (41.0 %) reported. Contact injuries were reported in 563 (40.6 %) patients, chemical burns in 128 (9.2 %) patients, radiation burns in 51 (3.7 %) patients, flame burns in 43 (3.1 %) patients, electrical burns in 17 (1.2 %) patients and friction burns in 16 (1.2 %) patients. Table 1 highlights the type of burn injury in patients under the age of 5, compared with the patients aged 5–16.

**Table 1:** Type of burn injury comparing patients aged <5 and patients aged 5–16

	All patients (*n* = 1387)	Patients aged <5 (*n* = 890)	Patients aged 5–16 (*n* = 497)	*p* value
Scald	569 (41.0 %)	374 (42.0 %)	195 (39.2 %)	0.333
Contact	563 (40.6 %)	397 (44.6 %)	166 (33.4 %)	0.001
Chemical	128 (9.2 %)	79 (8.9 %)	49 (9.9 %)	0.562
Radiation	51 (4 %)	15 (1.7 %)	36 (7.2 %)	0.001
Flame	43 (3.7 %)	9 (1.0 %)	34 (6.8 %)	0.001
Electrical	17 (1.2 %)	7 (0.8 %)	10 (2.0 %)	0.071
Friction	16 (1.1 %)	9 (1.0 %)	7 (1.4 %)	0.601

Peak prevalence for scald, contact and electrical burns was highest in the 1-year-old, chemical burns in the 2-year-old, flame burns in the 12-year-old, friction burns in the 2-year-old and radiation burns in the 15-year-old.

Table 2 highlights the differences in results that were reported in non-hospitalised compared with hospitalised patients. Of the patients requiring hospitalisation, there were a significantly higher number of reported scalds and flame burns, but a significantly lower number of contact burns radiation burns and chemical burns. The patients requiring hospitalisation were significantly younger, had a higher TBSA% and a higher rate of non-accidental injury.

**Table 2:** Results of hospitalised versus non-hospitalised patients

	Hospitalised patients (*n* = 356)	Non-hospitalised patients (*n* = 1031)	*p* value
Age	2 (1–4)	3 (1–9)	<0.001
TBSA%	1 (1–3)	1 (1–2)	<0.001
Scalds	179 (50.1 %)	390 (37.8 %)	<0.0001
Flame	23 (6.5 %)	20 (1.9 %)	0.0001
Contact	127 (35.7 %)	436 (42.3 %)	0.0287
Chemical	11 (3.1 %)	117 (11.3 %)	<0.0001
Electrical	5 (1.4 %)	12 (1.2 %)	0.7803
Friction	4 (1.1 %)	12 (1.2 %)	1.000
Radiation	7 (2.0 %)	44 (4.3 %)	0.0497

Median (IQR); number (%); TBSA: total body surface area

Table 3 highlights the mechanism of injury in both age groups for scalds, contact and chemical burns.

**Table 3:** Mechanism of injury in patients aged <5 and patients aged 5–16 for scalds, contact and chemical burns only

	All patients (*n* = 1260)	Patients aged <5 (*n* = 850)	Patients aged 5–16 (*n* = 410)	*p* value
Scald				
Hot beverage	334 (58.7 %)	248 (66.3 %)	86 (44.1 %)	0.001
Domestic water	168 (29.5 %)	94 (25.1 %)	74 (37.9 %)	0.020
Food item	67 (11.8 %)	32 (8.6 %)	35 (17.9 %)	0.006
Contact burn				
Portable household agent	254 (45.1 %)	175 (44.1 %)	79 (47.6 %)	0.083
Fixed household agent	241 (42.8 %)	190 (47.9 %)	51 (30.7 %)	0.001
Outdoor agent	61 (10.8 %)	28 (7.1 %)	33 (19.9 %)	0.004
Miscellaneous	7 (1.2 %)	4 (1.0 %)	3 (1.8 %)	0.706
Chemical				
Spill	47 (36.7 %)	21 (26.6 %)	26 (53.1 %)	0.008
Ingestion	25 (19.5 %)	20 (25.3 %)	5 (10.2 %)	0.139
Eye splash	56 (43.8 %)	38 (48.1 %)	18 (36.7 %)	0.670

Miscellaneous items: candle wax (2), man-hole cover (1), hot brick kiln (1), metal bar from bonfire (1), melted plastic (2)

There were 43 flame burns in total: 22 caused by playing with petrol, aerosols, a lighter or match, 20 caused by a child touching a flame (open fire, candle, bunsen burner, blow torch) and 1 teenager who was assaulted with a lighter. A total of 40 patients were recorded as having sunburn due to over-exposure, with 11 more reporting sunburn secondary to the use of a sunbed. Friction burns were caused by a treadmill in five cases, the seatbelt in a road traffic accident in four cases, five in playgrounds and two playing with ropes. Electrical burns were caused by damaged cables in four cases, touching plugs in sockets in eight, and children cutting through electrical cables in five cases.

The primary site of scald or burn is outlined in Table 4. The most commonly injured site in both age groups was a hand or digit.

**Table 4:** Primary site of scald and burn recorded in patients aged <5 and patients aged 5–16

Site	Patients aged <5 (*n* = 890)	Patients aged 5–16 (*n* = 497)	*p* value
Arm	104 (11.7 %)	88 (17.7 %)	0.003
Chest	92 (10.3 %)	22 (4.4 %)	0.001
Face	85 (9.6 %)	48 (9.7 %)	1.000
Neck/throat	8 (0.9 %)	3 (0.6 %)	0.755
Leg	69 (7.8 %)	69 (13.9 %)	0.001
Hand/digit	315 (35.4 %)	123 (24.7 %)	0.001
Foot/ankle	58 (6.5 %)	27 (5.4 %)	0.484
Other	159 (17.9 %)	117 (23.5 %)	0.0117

There were no recorded deaths in the patient cohort. A total of 356 (26 %) patients were admitted to hospital for further intervention, of which 117 (33 %) patients required hospitalisation for one or more days, with a median length of stay of 1 day (range, 1–28; IQR, 1–2).

## Discussion

Scalds and contact burns were the most commonly reported thermal injury in children aged less than 16 years, presenting to the ED of a regional burns unit for Wales and the South West England, over a 7-year period. The median age of the children in this study was 2 years, compared with a recent study in the UK that reported a mean age of 3.74 [1]. As in previous research, more male children presented to the ED with scalds and burn injuries than female [1, 2, 14]. Most scald and burn injuries occurred in the child’s own home in this study, which concurs previous research [1, 2].

Child maltreatment was suspected in only 1 % of cases which is lower than previously reported [1]. However, this result may be due to the inherent limitations in a retrospective study design. Deliberately self-inflicted burns were reported in 0.4 % of cases, all of which were older children using an aerosol spray, matches or lighter. A previous UK-based study discussed deliberate, self-inflicted burns and reported no such injuries in children under the age of 17 [14]. One case of assault by a teenager on another (using a lighter) was reported, although previous research in children is limited to make any comparisons.

As reported in previous research, the peak prevalence for scalds, contact and electrical burns was 1 year of age and the most commonly injured site was a hand or digit in both age groups [1]. The results of this study concur with those of Kemp et al. highlighting that the most common injury mechanisms in children involved a spill of a hot beverage or touching a hot object such as an oven hob, iron or hair straighteners [1]. In contrast to a number of previous studies; however [1, 2, 4, 5], contact burns were the most common burn type in children aged less than 5, with fixed household items being the most common causative agent, such as oven doors or hobs, central-heating components and various types of fireplaces and wood burners.

Previous burn prevention strategies have previously targeted these contact burn agents [9, 15]; however, portable devices such as hair straighteners also caused a large number of contact burns in children under the age of 5. Due to increasing popularity of hair straighteners over the last few years and the temperatures reached by these devices, it is imperative that innovations are needed to modify product design to reduce incidence of burns in children. A number of recent campaigns have been launched in the UK in an attempt to reduce contact burns caused by hair straighteners; however, the effectiveness of such campaigns is yet to be proven. Young children who have started to mobilise independently are commonly reported to suffer scalds due to knocking over hot beverages, and the results of this study supported this finding. As with contact burns, there has been a number of prevention strategies aimed at reducing scalds in children [16–18].

A meta-analysis of prevention strategies suggested that although home safety education, especially with the provision of safety equipment, is effective in increasing some thermal injury prevention practices, there is insufficient evidence as to whether this also reduces injury rates [19]. A more recent Cochrane review suggested that there is some evidence that burn intervention strategies may reduce injury rates, particularly where interventions are provided at home; however, further research is needed to confirm these findings [20].

The results from this study and previous research confirm that most paediatric burns occur in 1–2 years and are caused by hot liquids and fixed or portable household devices, in the child’s own home. Burns prevention strategies at a local level could include an educational video which is shown to parents prior to discharge home with a newborn baby. This is a strategy already used successfully in our health board for other education purposes. Devices such as lids for mugs and protective sleeves for hair straighteners could be made available to parents, especially considering the substantial costs that could be saved through the prevention of one severe burn alone.

The main limitation of this study was the use of the database to identify the patients for inclusion. It is possible that a number of patients were missed due to incorrect coding. The use of the database may have resulted in a degree of selection bias as errors may have occurred in the collation of the list of patients from the hospital database. A further limitation of the retrospective nature of the study is that not all of the medical notes could be successfully located; however, a rate of 1.6 % missing data was considered acceptable in a sample of this size. All data collected for each patient was retrieved from their ED medical notes; therefore, reliance was placed on the information being both accurately and legibly documented. Reliance on the ED notes for ascertaining non-accidental injury may also have led to a degree of inaccuracy, as this was recorded by the ED doctor based on suspicion alone and should be considered when interpreting the study results. Patients admitted directly to the burns unit would also have been missed leading to a degree of potential selection bias.

## Conclusions

The results of this 7-year retrospective study support previous findings that scalds and contact burns are the most common thermal injury in children aged less than 16. Common mechanisms were hot beverages, hobs and hair straighteners, highlighting further burn prevention strategies are needed and good-quality prospective studies that investigate the effectiveness of such strategies in reducing injury rates.
